# Comparing Low Volume Versus Conventional Volume of Polyethylene Glycol for Bowel Preparation during Colonoscopy: A Randomised Controlled Trial

**DOI:** 10.21315/mjms2023.30.5.9

**Published:** 2023-10-30

**Authors:** Muhammad Faeid Othman, Andee Dzulkarnaen Zakaria, Maya Mazuwin Yahya, Mohd Nizam Md Hashim, Wan Mokhzani Wan Mokhter, Wan Zainira Wan Zain, Ikhwan Sani Mohamad, Mohd Shahrulsalam Mohd Shah, Syed Hassan Syed Abd Aziz, Mohd Nasrullah Nik Ab Kadir, Zaidi Zakaria, Michael Pak-Kai Wong

**Affiliations:** 1 Department of Surgery, School of Medical Science, Universiti Sains Malaysia, Kelantan, Malaysia; 2 Department of Surgery, Hospital Universiti Sains Malaysia, Kelantan, Malaysia; 3 Endoscopy Unit, Hospital Universiti Sains Malaysia, Kelantan, Malaysia; 4 Department of Community Medicine, School of Medical Science, Universiti Sains Malaysia, Kelantan, Malaysia

**Keywords:** colonoscopy, polyethylene glycol, cathartics, patient satisfaction

## Abstract

**Background:**

Polyethylene glycol (PEG) solution is widely used as a colonoscopic bowel cleaning agent, although some patients are intolerant due to the need for ingesting large solution volumes and unpleasant taste. A low-volume solution may enhance patient tolerability and compliance in bowel preparation. Accordingly, this study compared the effectiveness of two difference PEG volumes for bowel preparation before colonoscopy in terms of bowel cleanliness, completeness of colonoscopy, patient tolerability and colonoscopy duration.

**Methods:**

Using a prospective randomised controlled single-blinded study design, 164 patients scheduled for colonoscopy were allocated to two groups (*n* = 82 patients in each) to receive either the conventional PEG volume (3 L, control group) or the low volume (2 L, intervention group). The Boston Bowel Preparation Scale (BBPS), a validated scale for assessing bowel cleanliness during colonoscopy, was used to score bowel cleanliness in three colon segments. Secondarily, colonoscopy completeness, tolerability to drinking PEG and the duration of colonoscopy were compared between the groups.

**Results:**

There were no statistically significant differences between the two intervention groups in terms of bowel cleanliness (*P* = 0.119), colonoscopy completion (*P* = 0.535), tolerability (*P* = 0.190) or the amount of sedation/analgesia required (midazolam, *P* = 0.162; pethidine, *P* = 0.708). Only the duration of colonoscopy differed between the two groups (longer duration in the control group, *P* = 0.039).

**Conclusion:**

Low-volume (2 L) PEG is as effective as the standard 3 L solution in bowel cleaning before colonoscopy; however, the superiority of either solution could not be established.

## Introduction

The overall incidence of colorectal cancer is increasing in Asia, especially in the rapidly developing countries of Southeast Asia, such as Malaysia (1). Colonoscopy is the preferred technique for evaluating the colonic and distal ileal mucosa for diagnosing and treating colorectal cancer (2). A recent survey reported that in the age group of 50 years old and above, about 6%–25% of people in European countries and about 62% in the United States of America have undergone colonoscopy within the past 10 years (3).

High-quality colonoscopy has been reported to be associated with favorable patient outcomes in colorectal cancer (4). Furthermore, the success of colonoscopy is linked to attaining cecal intubation and the adenoma detection rate, which directly corresponds to the quality of bowel preparation. To achieve good quality bowel preparation before colonoscopy, adequate cleansing of the colonic wall of the stool and staining fluid is needed (5), for which the two most widely used formulations are polyethylene glycol (PEG) and sodium phosphate (NaP) (2). While PEG is associated with a high incidence of nausea, vomiting and a bloating sensation, NaP is commonly associated with dizziness and biochemical abnormalities due to a high phosphate content of the solution (6). Despite the superior efficacy and safety of PEG compared to other bowel preparation agents, the requirement for the administration of large volumes of PEG to achieve the desired effect is its main disadvantage (7).

A few randomised controlled trials have evaluated the outcomes of bowel preparation by reducing the volume of PEG, but most of these trials have used an adjunct to substitute (8–12). Inadequate bowel preparation may affect both adenoma detection and cecal intubation rates. Poor bowel preparation is observed in about 20%–25% of colonoscopy procedures. Several predictive factors for inadequate bowel preparation have been documented, including patient compliance with instructions given during bowel preparation, chronic constipation, concomitant medication and lower socioeconomic status (13, 14).

The conventional PEG solution is widely accepted as a preparation agent for colonoscopy and has superior cleansing efficacy. Davis et al. (15) introduced the PEG electrolyte solution in 1980, which is an isotonic oral, non-digestible and non-absorbable solution. PEG ingestion does not result in electrolyte imbalance or acute renal failure; hence, it can be safely used in patients with critical conditions. However, the need for ingesting a large volume of fluid and the unpleasant taste restrict its popularity among patients (2, 16). Therefore, we conducted this randomised controlled trial to compare the effectiveness of two different volumes of PEG solution for colon preparation before colonoscopy.

## Methods

### Study Design

In this single-centre randomised single-blinded trial, we compared the efficacy of conventional volume PEG (3 L) with low volume PEG (2 L) for bowel preparation before colonoscopy.

### Study Sample

All potential subjects from the surgical outpatient clinic (SOPD) and general surgical ward (aged 18 years old and above) of the Hospital Universiti Sains Malaysia (HUSM), Malaysia, who were scheduled for colonoscopy, were invited to participate in this trial. Patients were excluded if colonoscopy was performed in the emergency setting, was a known or suspected case of gastrointestinal obstruction or perforation, toxic megacolon, had a history of colonic resection or relevant diseases that might interfere with the aim of the study.

### Sample Size

The sample size was calculated based on a dichotomous test using the Power and Sample Size software. The test design was set as an independent, prospective, two-proportions uncorrected chi-squared test. For testing two proportion populations, the reference value was based on the results reported by Harrison et al. (17), Lee SW et al. (13) and Menees et al. (14), who stated about 8% incidence (*P*_0_ = 0.08) of poor bowel preparation using the conventional volume PEG and about 25% (*P*_1_ = 0.25) with low volume PEG (2 L). Using a significance level (*α*) of 0.05 and a study power of 0.8, the sample size was calculated as 74 patients in each arm (total = 148). After considering a 10% dropout rate, the sample size was revised to 164 (*n* = 82 in each arm).

### Randomisation

Eligible subjects were randomly allocated to two groups: the control group, which received 3 L PEG (conventional volume) and the intervention group, which received 2 L PEG (low volume). All PEG volumes were administered in a split-dose manner. The conventional PEG volume (3 L) was consumed thrice: at 3 pm and 6 pm on the day before the colonoscopy and the last dose at 5 am early morning on the day of the colonoscopy. In the intervention group, patients were given PEG (2 L) twice: at 6 pm on the day before the colonoscopy and the final dose at 5 am on the day of the colonoscopy.

**Figure 1:**
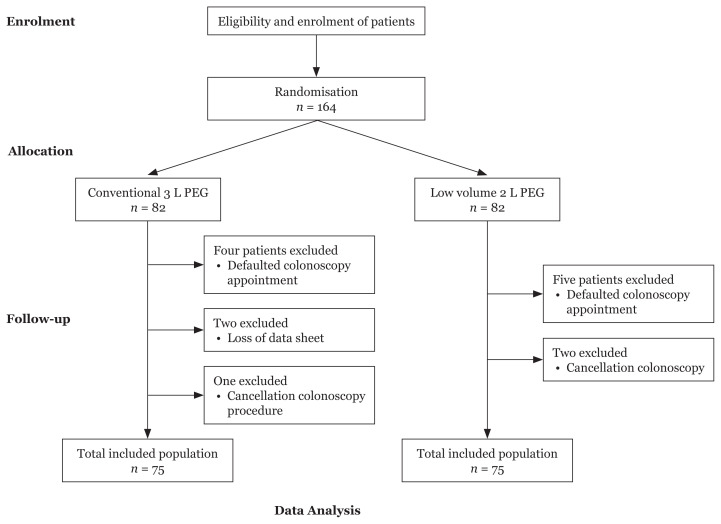
Flow chart of patients’ enrolment into the study

The grouping was done using conventional block randomisation. The researcher selected six blocks as the block size to carry out the randomisation—three blocks for each group. Using this strategy, 82 participants were randomly allocated to each group (Figure 1).

### Procedure

All eligible patients were screened based on the inclusion and exclusion criteria. Patients in the SOPD clinic or surgical ward who had planned to undergo elective colonoscopy were invited to participate and given a comprehensive explanation of the purpose and method of the study. Written consent was obtained from the patients who agreed to participate. Patient data, including full name, registration number, demographic data including age, weight, height, indication for colonoscopy and underlying disease, date of colonoscopy and the person taking consent, were collected before colonoscopy. All patient data were kept confidential and were used only by the investigators.

For this study, the endoscopists were also provided with a proforma to fill in the necessary information after the colonoscopy. The filled proforma information was also kept confidential. Since this was a single-blinded study, the endoscopists were blinded to the assigned treatments (i.e. they were unaware of the PEG preparations given to the participants undergoing colonoscopy). They were also prohibited from communicating with participants to ensure that confidentiality was maintained and did not cause any bias during the study.

### Assessment

The primary objective of this trial was to determine the effectiveness of different PEG volumes to achieve a clean colon before colonoscopy. For this purpose, we used the universal grading system, the Boston bowel preparation scale (BBPS), which measured the degree of cleaning in each segment of the large intestine, including the left-sided colon (rectum, sigmoid and descending colon), transverse colon and right-sided colon (ascending colon and caecum). Each segment was scored from 0–3: 0 = inadequate (unprepared colon segment with mucosa not seen due to solid stool that cannot be cleaned); 1 = poor (a portion of colon segment mucosa seen but other colon segment areas not adequately visualised due to tiny, residual stool particles and/or opaque liquid); 2 = good (minor residual tincture, small fragments of stool or opaque liquid remaining, but view is not very good); 3 = excellent (entire mucosa of colon segment seen well, with no residual staining, small fragments of stool or opaque liquid). In addition to the standard BBPS score, we used an additional score labeled ‘X’ to represent the state wherein the endoscope could not reach and visualise a specific segment of the bowel. We considered scoring ‘X’ as equal to 0 in BBPS. Adequate bowel preparation was defined as a cumulative BBPS score of ≥ 6.

Secondary outcome measures were the completion of colonoscopy procedure, tolerability toward PEG solution and duration of colonoscopy. A complete colonoscopy was confirmed when the caecum was visualised, which was characterised by the presence of *Taenia coli* convergence, appendicular lumen and ileo-cecal opening. Tolerability of the patient toward PEG solution was determined by identifying the capability of the patient to drink all amounts of PEG solution, as instructed. Finally, the duration of colonoscopy was measured starting from the time of entry of the endoscope until the scope was removed from the patient’s anus.

Statistical analyses were performed using SPSS version 26.0 (SPSS Inc., IBM Corp., Armonk, NY). The data were manually entered into the software before the cleaning process took place. Pearson’s chi-squared and independent samples *t*-tests were used to test the association between the subject characteristics based on the two different volumes of PEG. To evaluate the primary study objective, Pearson’s chi-squared test was used to analyse the association between the volume of PEG used with the total BBPS score and the ability of the scope to reach the cecal area. All the tests were two-sided and a *P*-value of < 0.05 was considered statistically significant.

## Results

A total of 164 eligible patients were randomised into two groups. Fourteen patients (seven from each group) were excluded for several reasons, such as defaulted colonoscopy appointments (3 L, *n* = 4; 2 L, *n* = 5), loss of patient data (3 L, *n* = 2) and late cancelation of the colonoscopy procedure (3 L, *n* = 1; 2 L, *n* = 2). Finally, 150 patients were included in the final analysis.

Table 1 summarises the descriptive characteristics of the patients. There were no significant differences between the two study groups regarding gender, weight, height, body mass index, the indication of colonoscopy, the experience of colonoscopy before and co-morbidities.

The majority of patients (*n* = 130, 86.7%) underwent colonoscopy performed by endoscopists with less than five years of experience, and the remaining 20 procedures (13.3%) were performed by endoscopists with more than 5 years of experience in this field. There were no statistically significant differences between the two study groups in this regard. The dosage of sedation and analgaesic used during colonoscopy was given based on the patient’s general condition and the tolerability of the colonoscopy procedure. The mean dosage of intravenous (IV) midazolam was 2.92 ± 1.07 mg and 31.4 ± 13.0 mg for IV pethidine. Both study groups were statistically comparable in terms of the dosage of sedation and analgesia (midazolam, *P* = 0.162; pethidine, *P* = 0.708).

A BBPS score in three different colon segments was added and the final BBPS score was used for analysis (good bowel preparation = score of ≥ 6). Table 2 shows that 62 patients (82.7%) in the 3 L (control) group and 54 patients (72.0%) in the 2 L (intervention) group successfully achieved adequate and good bowel preparation. There was no statistically significant difference between the two groups (*P* = 0.119).

A complete colonoscopy procedure is defined as the ability of the endoscope to show the most proximal part of the colon, the caecum. Table 3 shows 62 patients (82.7%) in the control group and 59 patients (78.7%) in intervention group had successfully completed colonoscopy procedure. There was no statistically significant difference between the two groups in the ability of the endoscope to reach the cecal area (*P* = 0.535).

Most of the patients (*n* = 140, 93.3%) were able to finish drinking the PEG solution as instructed; only 10 patients (6.7%) were unable to finish the solution as per the instructions. Both study groups were statistically comparable in terms of the tolerability of PEG (*P* = 0.19).

The mean duration of colonoscopy was 39.4 ± 19.3 min in the control group and 33.6 ± 14.0 min in the intervention group. This difference was statistically significant (*P* = 0.039) (Table 4).

## Discussion

Each sachet of PEG needs to be dissolved in 1 L of water for consumption. Despite being a safe and effective colon cleaning agent, the need to dissolve the PEG powder in plentiful water is one of the main factors leading to noncompliance in using PEG solution as a bowel preparation agent. At our centre, the standard conventional volume of PEG used for bowel preparation is three sachets dissolved in 3 L of water. In this trial, we aimed to compare the clinical outcomes with using a lower volume of PEG solution (2 L) versus the standard 3 L solution to achieve the desired colon cleaning effect. We observed that low-volume PEG was as effective in bowel preparation as the conventional PEG volume in terms of the degree of cleanliness of the colon. Several studies have also reported that 2 L PEG was able to attain equivalent colon cleanliness as conventional PEG volume with a 70% to 80% success rate; however, most of these studies have compared 4 L and 2 L PEG with adjunct medications like simethicone and ascorbic acid (11, 18–20).

In this study, PEG administration was standardised using a split-dose regimen. A split-dose intake of PEG provides the most effective bowel cleansing. It has been established that preparations with split-dosage PEG provide a significantly better quality of colon cleansing than those with a non-split dosage, regardless of fluid volume. Furthermore, recent data confirmed that the sooner the procedure is performed since the last fluid intake, the higher the chance of obtaining an adequate level of bowel cleansing. Colonoscopy performed within 6 h–8 h of completion of bowel preparation is associated with better quality than a colonoscopy performed after 8 h. Beyond this time limit, the advantages of split dosage on the extent of the cleaning process are diminished (12, 21).

Several factors influence the successful completion of colonoscopy. It is known that colonoscopy performed on a symptomatic patient has a low success rate of reaching the cecal area when compared to colonoscopy performed on an asymptomatic patient (surveillance or screening) (22). An incomplete colonoscopy may ensue because of an abnormality within the colon that interferes with the scope’s pathway to reach the cecum. The endoscopist’s skills and performance are other essential elements in determining the success of the procedure (22). Endoscopists with adequate experience in dealing with difficult colonoscopy for many years tend to successfully complete the procedure compared to the newly practicing endoscopists. In our trial, the results for successful completion of colonoscopy were comparable to those of previous studies (10, 22–24). The majority of patients included in our study underwent colonoscopy for symptomatic reasons and patients with a history of bowel-related surgery were already excluded from this research. Most of the colonoscopies were performed by trainee endoscopists with less than 5 years of experience in endoscopic procedures. In most cases, incomplete colonoscopy resulted from poor bowel preparation (blockage by faeces), acute angulation of the colon and obstruction by a tumor or mass.

Tolerability and compliance with instructions are other factors relevant to predicting good bowel preparation. The patient’s ability to drink the whole of the instructed amount of PEG is crucial in ensuring that the colon is well prepared before the colonoscopy procedure. In our study, there was no significant difference in the proportion of patients who could tolerate the administered amount of PEG in either study group. Interestingly, a few previous studies concluded that most patients who drank a standard large volume of PEG had issues pertaining to tolerability and compliance with this solution (25, 26). To ensure compliance in our study, patients were explained in detail about colonoscopy procedure and encouraged to drink the full amount of solution as instructed. In addition, some of the patients were reminded via phone to ensure that all instructions were followed; consequently, higher compliance rates were achieved.

The duration of colonoscopy is measured from the moment of introducing the scope until its retrieval from the anus. In our study, the duration of the procedure was slightly longer in the control group compared to the intervention group. The possible reason for this difference is due to the timing used to clear up all the residual staining liquid in the colon while introducing the scope toward the proximal colon. Other possible factors that may affect the duration of colonoscopy are the quality of bowel preparation, the experience of endoscopists and conducting additional procedures, such as a biopsy and polypectomy (27). Furthermore, both obese and skinny patients are considered a challenge in performing colonoscopy. The increase in bowel looping and angulation in a lean patient or the difficulty in carrying out special maneuvers in an obese patient contributes to prolonging the duration of colonoscopy (27).

The strengths of this study are its study design (prospective randomised control trial) and its strategy to ensure randomised patient allocation to the study groups. In addition, blinding the endoscopist to group allocation ensured that there was no opportunity for bias in assessing the quality of bowel preparation. However, the core limitation of this study was the small sample size, which limits the application of our results to a broader population. In addition, since the study was conducted in a single tertiary care centre, there may be a centripetal population bias. Therefore, a more extensive population sampling involving multiple centres is recommended to confirm our results.

## Conclusion

A low-volume (2 L) PEG solution is as safe and effective as the conventional 3 L solution for bowel cleaning before colonoscopy. However, our study could not demonstrate the superiority of either group in terms of the effectiveness of cleanliness, successful completion of the colonoscopy, and tolerability and compliance with PEG.

## Figures and Tables

**Table 1:** Descriptive characteristics of the patients in the study (*N* = 150)

Clinical characteristics	*N* = 150 *n* (%)	Volume of PEG, *n* (%)		*P*-value

		3 L PEG *n* = 75	2 L PEG *n* = 75	
Endoscopist				0.631^a^
< 5 years experienced	130 (86.7)	64 (85.3)	66 (88.0)	
> 5 years experienced	20 (13.3)	11 (14.7)	9 (12.0)	
Sex				0.621^a^
Male	85 (56.7)	41 (54.7)	44 (58.7)	
Female	65 (43.3)	34 (45.3)	31 (41.3)	
Age, mean (SD)	55.9 (15.5)	54.6 (16.0)	57.3 (15.0)	0.282^b^
Weight, mean (SD)	61.9 (10.9)	62.5 (12.2)	61.3 (9.5)	0.502^b^
Height, mean (SD)	160.5 (7.3)	159.5 (8.2)	161.5 (6.3)	0.087^b^
BMI, mean (SD)	24.0 (3.9)	24.5 (4.0)	23.5 (3.8)	0.128^b^
Duration				
Colonoscopy, mean (SD)	36.5 (17.1)	39.4 (19.3)	33.6 (14.0)	0.039^b^
Dose of midazolam, mean (SD)	2.92 (1.1)	3.04 (1.15)	2.79 (0.99)	0.162^b^
Dose of pethidine, mean (SD)	31.4 (13.1)	31.8 (13.8)	31.0 (12.3)	0.708^b^
Indication for colonoscopy				0.132^a^
Symptomatic	132 (88.0)	63 (84.0)	69 (92.0)	
Screening/Surveillance	18 (12.0)	12 (16.0)	6 (8.0)	
Previous colonoscopy experience				0.597^a^
Yes	16 (10.7)	7 (9.3)	9 (12.0)	
No	134 (89.3)	68 (90.7)	66 (88.0)	
Comorbidity				0.618^a^
Yes	61 (40.7)	29 (38.7)	32 (42.7)	
No	89 (59.3)	46 (61.3)	43 (57.3)	

Note:

a Pearson’s chi-squared test;

b Independent *t*-test

**Table 2:** Association between volume of PEG and Boston Bowel Preparation Scale (*n* = 150)

	Boston Bowel Preparation Scale (BBPS)		*χ*^2^ stat (df)	*P*-value^a^

	Poor (5 and below) *n* (%)	Good (6 and above) *n* (%)		
Volume of PEG				
3 L PEG	13 (17.3)	62 (82.7)	2.43 (1)	0.119
2 L PEG	21 (28.0)	54 (72.0)		

Note:

a Pearson’s chi-squared test

**Table 3:** Secondary endpoints between the two groups (*n* = 150)

		3 L	2 L	*χ*^2^ stat (df)	*P*-value a
Completeness of colonoscopy	Yes	62 (82.7)	59 (78.7)	0.39 (1)	0.535
	No	13 (17.3)	16 (21.3)		
Tolerability to drink completely PEG	Yes	68 (90.7)	72 (96.0)	1.71 (1)	0.190
	No	7 (9.3)	3 (4.0)		

Note:

a Pearson’s chi-squared test

**Table 4:** Numerical secondary endpoint between two groups (*n* = 150)

	3 L	2 L	*P*-value *
Duration colonoscopy (mean, SD)	39.4 (19.3)	33.6 (14.0)	0.039

Note:

* Independent *t*-test
